# Optimization of Heart Failure Patients Discharge Plan in Rajaie Cardiovascular Medical and Research Center: An Action Research 

**DOI:** 10.30476/ijcbnm.2021.87770.1461

**Published:** 2021-07

**Authors:** Fidan Shabani, Farahnaz Mohammadi Shahboulaghi, Nahid Dehghan Nayeri, Mohammadali Hosseini, Majid Maleki, Nasim Naderi, Mohammad Chehrazi

**Affiliations:** 1 Rajaie Cardiovascular Medical and Research Center, Iran University of Medical Sciences, Tehran, Iran; 2 Iranian Research Center on Aging, University of Social Welfare and Rehabilitation Sciences, Tehran, Iran; 3 Nursing and Midwifery Care Research Center, School of Nursing and Midwifery, Tehran University of Medical Sciences, Tehran, Iran; 4 Department of Nursing, University of Social Welfare and Rehabilitation Sciences, Tehran, Iran; 5 Department of Biostatistics and Epidemiology, School of Public Health, Babol University of Medical Sciences, Babol, Iran

**Keywords:** Action research, Discharge planning, Heart failure, Hospitalization

## Abstract

**Background:**

Chronic heart failure can lead to frequent hospitalizations. Improving the discharge planning is an approach to reduce hospitalization. Since there has not been enough structured and effective discharge plan in Iranian hospitals, the present study was designed to optimize this program.

**Method:**

This is a participatory action research based on Hart and Bond’s framework, conducted in a cardiovascular center in Iran from June 2016 to April 2018 during two cycles. Based on the optimization strategies obtained through semi-structured interviews with 15 participants, three focus group discussions and six expert panels, the operational discharge plan, including three areas of patient empowerment, telephone follow-up and home visit, was designed, implemented for three months and evaluated for 23 patients. European Heart Failure Self-Care Behavior Scale and information registration form to record the number of hospitalization and length of hospital stay were used to collect the quantitative data. The non-parametric Wilcoxon test was used to analyze the data by SPSS 16. Qualitative participatory evaluation was performed during a group discussion and analyzed based on qualitative content analysis method with conventional approach P<0.05 was statistically significant.

**Results:**

Considering the solutions provided by the participants, the operational discharge plan was designed and implemented with the cooperation of relevant stakeholders. Evaluation showed significant effects of designed discharge plan on self-care behavior (P<0.001), number of hospitalizations (P<0.001), and length of hospital stay (P<0.001).

**Conclusion:**

Changes were made to improve the heart failure patients’ discharge plan using action research, which resulted in reduced re-hospitalization and improved self-care behavior.

## INTRODUCTION

Heart failure is the leading cause of death due to cardiovascular problems and its incidence and prevalence are increasing. ^1^ Approximately 6.5 million people in the United States have heart failure, and the annual cost of caring for these patients is estimated $39 billion. ^2^ The prevalence of heart failure in developed countries is about 1-2% and its prevalence is also high in developing countries. For example, the prevalence of heart failure in Iran is shown to be approximately 3.5% in the near future. ^3^ Chronic heart failure can lead to frequent hospitalizations and reduced life expectancy. ^4^ About 20-25% of patients are re-hospitalized within a month of discharge. ^5^ Heart failure is one of the most common reasons for re-hospitalization in the first 60 days after discharge from hospital. ^6^ Inadequate planning for discharge from hospital and non-compliance with treatment of patients lead to re-admission of patients with heart failure, and this shows the importance of discharge plan in improving the patients’ quality of life. ^7^

Ways to reduce re-admission of patients with heart failure include the use of a discharge plan, patient education, and post-discharge support. ^8^ An effective discharge plan significantly improves a person’s health and reduces readmission. ^9^ The goals of the discharge plan are to continue quality care between the hospital and community, to reduce the length of hospital stay and re-hospitalization and improve post-discharge coordination services. ^10 , 11^

The emphasis on discharge planning varies from country to country. In the United States, having a discharge planning is mandatory for hospitals. ^12^ Although the discharge plan is an accepted activity in the United States hospitals, there is no global or accepted operational model for the discharge planning process. ^13^ In many health systems today, the use of a standard discharge planning process is considered as an indicator of quality of health care and essential for health care systems. ^14 , 15^

Recent studies have shown that there is no structured and effective discharge plan in Iranian hospitals. ^16^ Implementation of a discharge plan in Iran is associated with various challenges. Strategies for creating and upgrading discharge plans in Iran should be implemented with the help of various policies, programs and support laws. ^15^ The implications for discharge plan implementation are not yet clear in Iran and more research is needed. ^17^

The first version of heart failure patients discharge plan of Rajaie Cardiovascular, Medical and Research Center in Tehran was developed in June 2015 by the health education unit. The product of this plan was a two-page pamphlet in which the education of patients with heart failure was referred to. This education was implemented by nurses at the patient’s bedside individually and face to face on the day of discharge. The discharge plan received some criticisms. For example, discharge training was provided on the day of discharge when the patient was not focused enough to learn due to discharge anxiety. Also, reducing the length of hospital stay and readmission of these patients were a great concern for hospital physicians and nurses, so there was a need to modify the discharge plan. 

To address this issue, the current study was designed to optimize the discharge planning for heart failure patients. Action research can be highly effective for achieving organizational change in healthcare by engaging managers as well as practitioners in the change process. ^18^ Therefore, this study was conducted with an action research approach so that by involving the patients, healthcare professionals and hospital managers in optimization process of discharge plan, we can design programs based on heart failure patients’ needs in Iran and make a positive change in the discharge process of these patients in our country.

## MATERIALS AND METHODS

This is a participatory action research based on Hart and Bond (1995) framework and used a combination of qualitative method with content analysis approach and quantitative methods. The Hart and Bond Action Research Framework includes six steps: 1. Reflect on a theme, 2. Plan action, 3. Take action to change Practice, 4. Observe and evaluate, 5. Reflect again, and 6. Plan further action. Action research is being used in many settings and can be very effective in healthcare organizations. ^18^

The study was conducted at Rajaie Cardiovascular, Medical and Research Center in Tehran, which is one of the largest cardiovascular hospitals in Asia and a referral center for cardiovascular disease across the country, from June 2016 to April 2018. This hospital has 606 active beds. An average of 22,000 patients are admitted to this center annually. One of the departments of Adult Cardiac Care is “Heart Failure and Cardiac Transplantation Department” and many heart failure patients refer to this center from all over the country. This center has five Cardiac Care Units (CCU) and most of the heart failure patients are admitted in these units. Action research was conducted in these five units of the hospital. Three of these units have 12 beds and one has 21 beds. In these 4 CCUs, patients are admitted with different heart problems including heart failure. In the other CCU of the hospital, 7 beds are allocated to heart failure patients only. 

The researcher (F.Sh) worked as a nurse in one of the CCUs of this Hospital for 12 years, had experience in caring for patients with heart failure, and facilitated the project. The researcher’s efforts, as a link between researchers and healthcare professionals, led to progress of the project and facilitated the change.

Continuous learning during action research maximized the collaboration between the participants and facilitated the designed discharge plan implementation. In this study, two cycles of action research were implemented. The discharge plan was very well received by patients, caregivers and heart failure specialists, and hospital officials were eager to continue the program.

First cycle, reflect on a theme: In the first stage of this cycle to explain the problem, the main researcher focused on the critical problem of the Cardiac Care Units of hospital where she worked. She had 12 years of experience as a nurse in CCU. Based on the Rajaie hospital strategic plan, high readmission rate of the heart failure patients was a threat for hospital resources.

Two focus group discussions, each lasting 60 minutes, were conducted as a qualitative study with content analysis approach and conventional content analysis as the data analysis method. Data were collected about the existing problem and its causes based on the perceptions and actual experiences of the health care providers of the desired wards through purposeful sampling. Participants were CCU nurses (n=4), cardiologists (n=3), and nursing supervisors (n=3). The nurses’ inclusion criteria were having a bachelor’s degree in nursing or higher, having at least 5 years of work experience with a heart failure patient, having consent to participate in the study. The cardiologists’ inclusion criteria were having a heart failure fellowship and consent to participate in the study. The main question was “what are the main challenges of heart failure patients in the ward?” Besides that, the statistics of 48 heart failure patients who had been re-hospitalized in one of the CCUs were obtained based on the information recorded in the hospital during the last 3 months. 

Plan action: In the second stage of the first cycle, in order to find solutions to optimize discharge plan, we conducted a qualitative study and the solutions were obtained in semi-structured interviews with 15 participants, including 7 patients, 4 nurses, 2 cardiologists and 2 family caregivers. At this stage, purposive sampling continued based on the inclusion criteria until data saturation was reached. Duration of interviews was at least 33 minutes, and the maximum was 67 minutes.

Patients’ inclusion criteria were hospitalization with decompensated heart failure, current hospitalization less than 30 days prior to the last discharge, age of 18 years or older, and consent to participate in the study. Family caregivers’ inclusion criteria included: being the patient’s main caregiver, at least 6 months of patient care, and consent to participate in the study. The previous inclusion criteria were considered for nurses and cardiologists. Exclusion criteria were the participant’s unwillingness to continue participating in the study.

Interview questions were developed to clarify the actual and potential solutions and its facilitators. Different questions were designed for participants, for example, “based on your experience please explain what you need to know and do after discharge from hospital, to manage your disease effectively?” (Patients) or “after discharge, what are your main problems to take care of your patient at home?” (Family caregivers). ”what can you do for your patients to have less readmission?” or “based on your experiences with patients with frequent readmission, please explain what they need to know and do before and after hospital discharge?”(Cardiologists and nurses).

In order to analyze the qualitative data, we used qualitative content analysis method with conventional approach. To analyze the content, we used three steps: preliminary, organizing and reporting the analysis process and the results. ^19^ Semantic units were extracted from the text of each interview and coding was performed. Then, due to the similarities and differences, the codes were placed under the primary categories. Then, the sub-categories were combined, and the main categories were extracted. MAXQDA 10 software was used to manage the data.

Then, a focus group discussion was held for two hours with the presence of the researcher, four nurses, four doctors, five supervisors, five head nurses and three hospital managers. In this session, the extracted strategies and solutions from the last interviews were presented. Then, the strategies were prioritized by the participants, and the main areas of the discharge plan for improvement were identified. Prioritization was based on Suitability, Feasibility, and Flexibility (SFF) matrix. Each criterion (Suitability, Feasibility and Flexibility) can score 1 to 3, so each solution can have a score of 3 to 9. ^20^ The researcher asked the participants of this step to rate every solution from 3 to 9. 

After that, based on the suggestions of the participants and systematic literature, the action plan was developed by holding six two-hour expert panels with the presence of researcher, four nurses, two doctors, two supervisors, two hospital managers, and one executive manager. In designing and setting up the operational plan, recording the duties and activities of each participant and the time allocated to each activity, resources and facilities, costs, and evaluation of each activity were considered. The designed discharge plan reflected the results of interviews, focus group discussions and expert panels.

Take action to change practice: In the first step of the third stage of this cycle, working groups were formed based on the purpose of each area of the action plan to provide the required infrastructures. They included adjusting and allocating the nursing staff for home visits, preparing valid educational materials for patients and their family, designing of home visit assessment tools for patients after discharge, assigning a patient counseling hotline and ambulances for home visit, designing heart failure discharge planning flowcharts, establishing a home visit center and a patient counseling and education office. A 13-day workshop was held for nurses participating in the study and they got familiar with principles of heart failure, nursing care, treatment, patient education, and empowerment. Also, a 6-day home visit workshop was held for the same nurses. Then, the designed discharge plan was started and conducted for one month for 23 patients who were 18 years or older, hospitalized with the diagnosis of advanced heart failure, with Left Ventricular Ejection Fraction ≤30%, having the class III-IV symptoms of heart failure according to New York Heart Association classification, and hospitalization experience more than once in the last 6 months. All of them lived in Tehran and signed their consent form to participate in the program. 

For implementation of the patient empowerment area of discharge plan, initial assessment of the patient’s condition based on patient assessment form was performed in the first 24 hours of hospitalization by nurses. If the patient was hemodynamically stable, three consecutive training sessions (30 minutes for each session) were performed for 3 consecutive days in the hospital by the nurses participating in the program using the teach back method; then, the learning outcome was evaluated by a checklist and the training was repeated, if necessary. The day after discharge, the discharge nurse contacted the patient and arranged a home visit for the next 24 to 48 hours. The number of sessions and intervals of home visits were determined according to the patient’s condition and the opinion of the treating physician. The patient’s telephone follow-up was performed every 3 days by the nurses participating in the study to continue the training, follow the treatment, and be aware of the patient’s condition. It was also possible for the patient to contact the care team 24 hours a day. 

Observe and evaluate: In the fourth stage of the first cycle, that is one month after the start of the designed discharge plan, process evaluation was performed both quantitatively and qualitatively. Qualitative participatory evaluation was conducted during a group discussion session with the presence of the researcher, four nurses, two doctors, three patients, two family caregivers and two hospital managers. Participants were asked to explain about the positive and negative outcomes of the program. Group discussions were recorded and transcribed. Then, the researchers analyzed the transcripts, identified the quotations made by participants, and fixed the themes that had emerged during the discussions. In order to analyze qualitative data, qualitative content analysis method with conventional approach was used. Quantitative evaluation was performed by comparing the number of hospitalizations and length of hospital stay, 30 days before and 30 days after the implementation of the discharge plan. The Wilcoxon nonparametric test was used to analyze the quantitative data.

Reflect again: Then, in the fifth stage of this cycle, a group discussion session was held with the presence of researcher, four nurses, two doctors, two patients, two family caregivers, and two hospital managers. Results were reviewed and exchanged, and the participants expressed their views and obtained results from the evaluation stage. The researchers asked them to provide feedback on all discharge plan components. The main question was “is any change needed and what is your advice for improvement?”

**Figure 1:**
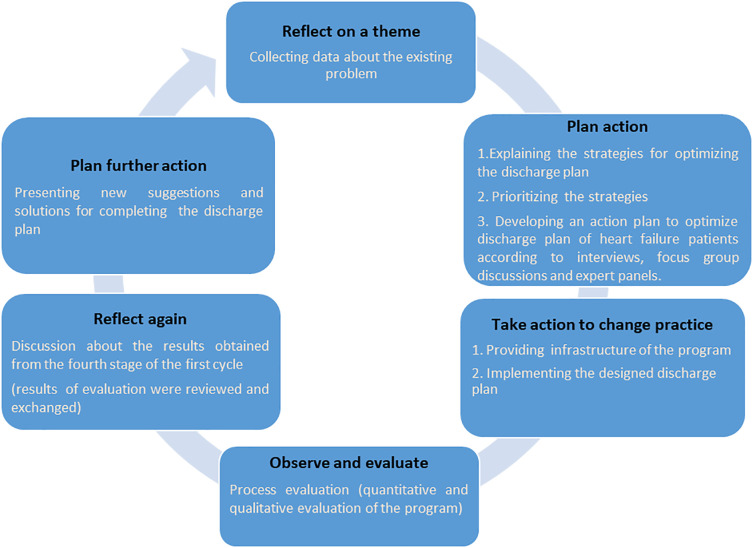
The first cycle of the action research process

Plan further action: In the sixth stage of the first cycle, based on the results obtained from the fifth stage, in a group discussion session, new suggestions and solutions for completing and reviewing the discharge plan were presented by the same participants in the fifth stage. The first cycle of the action research process is presented in Figure 1. 

Second cycle, reflect on a theme: This cycle started after receiving feedback from the first cycle and the steps were the same as the first cycle. In the first stage of the second cycle, the problem was redefined in a discussion session, based on the feedback of the first cycle, and the participants’ responsibilities for change were identified.

Plan action: In the second stage of this cycle, reprogramming was performed for the problems raised in the implementation of the operational plan. The first and second stages of this cycle were conducted with the presence of the researcher, four nurses, two doctors, one supervisor, and two hospital managers. 

Take action to change practice: The third stage of the second cycle continued for two more months for the same 23 patients with the implementation of the operational plan by applying new changes. 

Observe and evaluate: In the fourth stage of the second cycle, the final evaluation of the program was performed using quantitative methods. For this purpose, the self-care behavior, number of hospitalizations, and length of hospital stay for heart failure patients were compared before and three months after implementation of the discharge plan. At this stage, the data were collected using the European Heart Failure Self-Care Behavior Scale (EHFScB) and information registration form to record the number of hospitalization and length of hospital stay. The 12-item EHFScB was developed and tested to measure the patient’s self-care behaviors. In a psychometric study, data from 2592 heart failure patients from six countries were analyzed. Validity was established by interviews with heart failure experts and patients, item analysis, confirmatory factor analysis and analysis of the relationship between the EHFScB scale and scales measuring the quality of life and adherence. Internal consistency of the 12-item scale has been reported equal to 0.77 by the Cronbach’s alpha coefficient method. ^21^ This scale includes 12 items based on a 5-point Likert scale ranging from 1 (strongly agree) to 5 (strongly disagree). The total score was considered, and a lower score showed better self-care. In a study in Iran, the content validity index (CVI) was confirmed by an expert panel (CVI=0.97), and its reliability was assessed by Cronbach’s alpha (α=0.74). ^22^

The Wilcoxon nonparametric test was used to analyze the quantitative data. The significance level was considered to be less than 0.05 and analyses were performed in SPSS software version 16 (SPSS for Windows, Version 16.0. Chicago, SPSS Inc.).

Reflect again: In the fifth stage of this cycle, a group discussion session was held in the presence of the researcher, four nurses, two doctors, one supervisor, three patients, two family caregivers and two hospital managers ,and the results were reviewed and exchanged; then, the participants expressed their views on the results. 

Plan further action: In the sixth stage of the second cycle, the results obtained from the previous stage were presented in a group discussion session with the presence of the same participants in the fifth stage. Then, new suggestions were presented to continue the discharge plan. Finally, with the implementation of two action research cycles, the discharge plan of heart failure patients was improved.

**Figure 2:**
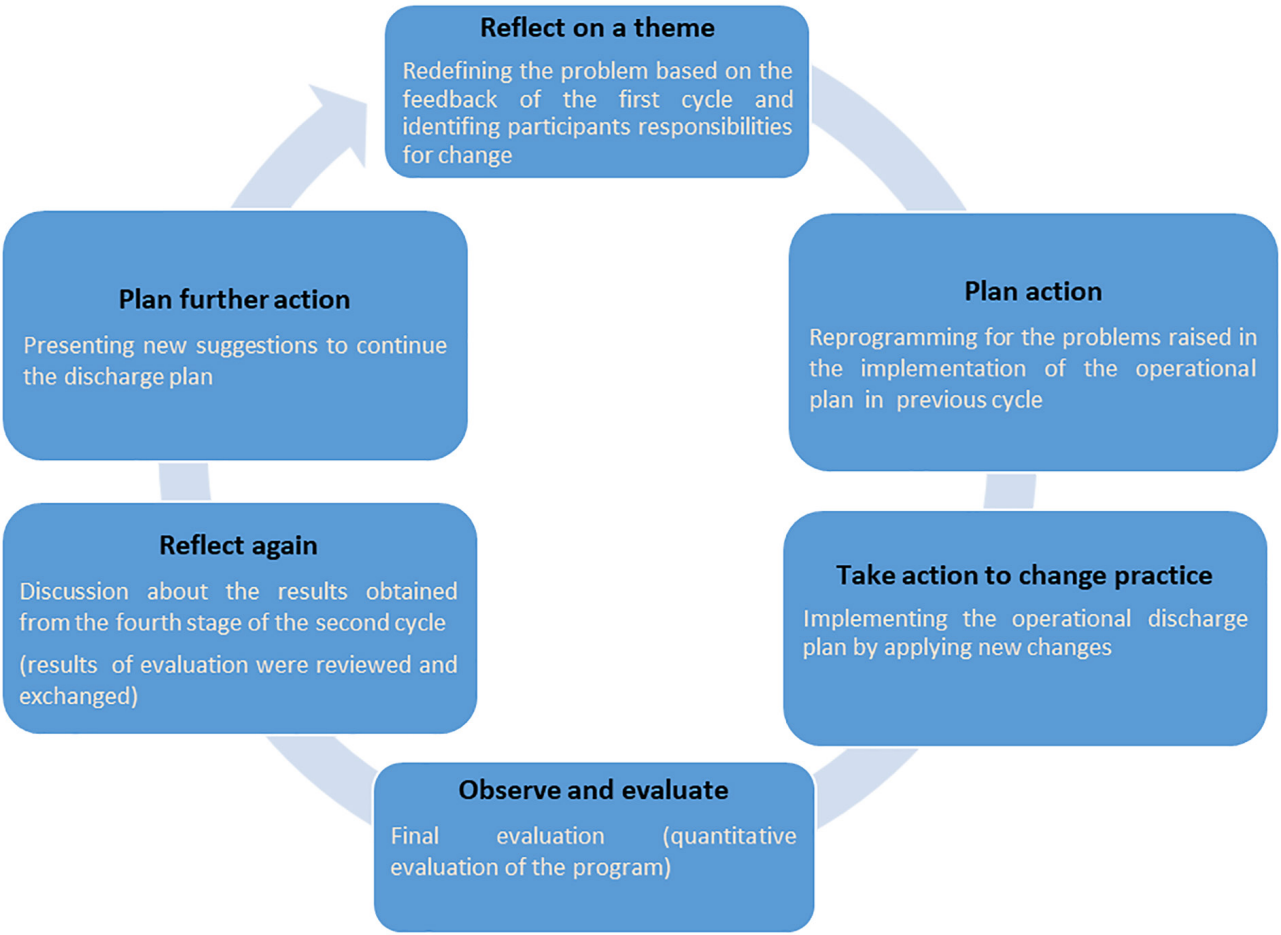
The second cycle of the action research process

Herr and Anderson (2005) criteria were used to achieve validity in this action research. To assure democratic validity, relevant stakeholders were identified and invited to participate in the study. Collaboration among stakeholders facilitated the change in the context. Improvement in the patients’ outcomes and also empowerment of healthcare professionals in different aspects of heart failure management assured the outcome validity. To assure process validity, the researchers used the triangulation method to collect data including interview, focus group discussion and panel of experts. The findings of the study were also continuously discussed with the research team. At the end of each group discussion, the topics discussed were checked in a summary with the participants, and the findings and steps of the work were recorded in a detailed and auditable form. The second cycle of the action research process is presented in Figure 2. 

This study was approved by the Ethics Committee of the University of Social Welfare and Rehabilitation Sciences (IR.USWR.REC.1395.89). After obtaining permission from the hospital authorities and identifying eligible participants, informed and written consent was obtained from the participants to take part in the study and recording of interviews with emphasis on the confidentiality of information and the non-use of the participants’ names at all stages of the research. The participants were assured that they had the right to withdraw from the study at any stage of the study. 

## RESULTS

Findings of the first cycle: In the first stage, based on the evaluation of the profiles of 48 patients admitted in a quarterly period, in terms of re-hospitalization in the last 30 days, it was found that 58% of patients had experienced re-hospitalization in less than 30 days after the last discharge from hospital. It means readmission of patients with heart failure is a common high-rate problem. Content analysis of the focus group discussions also confirmed the existence of a challenge in heart failure readmission. Predominant themes were frequent hospitalizations, non-compliance with treatment, lack of patient’s health literacy, complex nature of heart failure, failure in patient training, failure of discharge follow up system, and defective patient social security. This stage showed that the heart failure department of the hospital needed an effective discharge plan. 

Most participants in the first step of the second stage in the first cycle (60%) were women and their age was between 27 and 65 (47.40±11.15). Nurses had at least 5 years of experience with heart failure patients. Based on semi-structured interviews, 573 initial codes, 19 sub-categories, and 4 main categories related to discharge plan optimization strategies were extracted. Findings are presented in Table 1. 

**Table 1:** Sub-categories and main categories of the strategies for optimizing the discharge plan for heart failure patients (Results of qualitative content analysis of the first step of the second stage in the first cycle)

Sub-categories	Main categories
Maintaining contact with the patient after discharge	Telephone follow-up of patients after discharge
Checking the compliance with the treatment by phone	
Increasing the attractiveness of the training	Patient empowerment
Using new teaching methods	
Educational content to promote treatment adherence	
Continuous patient training	
Self-management training	
Motivating to continue treatment	
Therapeutic communication in patient education	
Understandable training for the patient	
Changing the patient’s attitude towards the disease	
Educating based on patient’s needs	
Determining how to follow the patient at home	Home visits after discharge
Establishing the possibility of the presence of the health team at home
Family-centered discharge program
Strengthening insurance coverage	Strengthening public coverage of health services
Strengthening the support of non-governmental organizations	
Strengthening financial support systems	
Increasing patients’ access to specialized/sub-specialized medical centers in all cities	

The solutions and strategies were prioritized according to SFF matrix and the mean score of participants for each area was: “Patient empowerment” 8.36±0.63, “Telephone follow-ups after discharge” 6.81±1.22, “Home visits after discharge” 5.77±0.66, and “Strengthening public coverage of health services” 4.95±0.47. Based on the agreement of the participants, it was decided that the solutions that have a high score in prioritization (with an average of more than 5) should be considered as the main areas of the discharge plan and will reach the operational stage. Therefore, the first three areas including “Patient empowerment”, “Telephone follow-ups after discharge”, and “Home visits after discharge” reached the operational stage. Steps and details of each area of the designed discharge plan are provided in Table 2. 

**Table 2:** Discharge plan for heart failure patients: Areas, steps and detailed activities

Areas of Discharge Plan	Steps of Discharge Plan	Detailed activities
Patient empowerment	Identifying patients’ educational needs	•Carrying out a qualitative study and determining the strategies for optimizing the discharge plan
		•Preparing a complete list of educational needs of patients with heart failure based on the needs assessment form
	Development of appropriate educational content (educational booklet)	•Determining the main topics of patient education based on literature review and extracted strategies and solutions from the interviews
		•Preparation of appropriate education content in the form of patient’ education booklets
	Preparation of infrastructure for patient empowerment program	•Determining the topics of the nurses’ training workshop for patient empowerment
		•Provision of appropriate educational content by workshop professors
		•Determining the time and place of the workshop
		•Selection of nurses participating in the nurses’ training workshop to empower the patient
		•Holding a training workshop for nurses to empower the patient
		•Preparation of initial patient assessment form in the hospital
	Implementation of a patient empowerment program in the hospital	•Initial assessment of the patient’s condition based on patient assessment form by nurses
		•Conducting three consecutive training sessions (30 minutes for each session) for 3 consecutive days in the hospital by the nurses participating in the program using the teach back method
		•Evaluation of the learning outcome by checklist
		•Repeating the training if necessary
Telephone follow-up after discharge	Preparing telephone follow-up infrastructure for patients	•Providing hot line and mobile phones to follow patients
		•Preparation of patient telephone follow-up form
	Making it possible for the patient to contact the nurse	•Providing the telephone number of the discharge nurse to the patient prior to discharge
		•Guiding the patient if the patient calls the discharge nurse at any time of the day or night
	Carrying out telephone follow-up of patients with heart failure	•Contacting the patient or patient’s family and completing the patient follow-up form by discharge nurse, 24 to 48 hours after discharge
		•Contacting the patient every 3 days to check the patient’s condition and performing a telephone triage
Home visits after discharge	Preparing infrastructure for implementing a home visit	•Establishment of a home visit center and a patient counseling and education office
		•Assigning a patient counseling hot line
		•Assigning ambulances and transportation for home visit
		•Adjustment and allocation of nursing staff for home visits
		•Providing the necessary equipment for home visit
		•Designing home visit assessment tools for patients after discharge
		•Holding a home visit workshop for nurses who participated in the patient empowerment workshop
	Performing home visits after discharge	•Contacting the patient the day after discharge, and arranging a home visit for the next 24 to 48 hours.
		•Determining the number of sessions and intervals of home visits according to the patient’s condition and the opinion of the treating physician
		•Checking the patient’s condition, recording vital signs, determining the degree of adherence to the treatment, interviewing the patient and family to identify problems, educating the patient and family in each home visit, also determining that the patient is in the green, red, or yellow zone of ​​heart failure and treating according to guidelines and cardiologist’s order

In the third stage, the discharge plan was implemented for one month. In the first month of the program, 28 home visits and 106 telephone follow-ups were performed for two thirds patients. Among the study cases (n=23), in the quantitative stage of the program, of the patients surveyed were male higher percentage were 61 to 70 years old; the education level of more than half of the patients was below the diploma. The majority of patients were married and most of them lived with their family. Higher percentage of the patients had ejection fraction of 10 percent. The results are presented in Table 3.

**Table 3:** Demographic characteristics of patients who participated in quantitative stage of the program (n=23)

Demographic Variable		N (%)
Sex	Female	6 (26.10)
	Male	17 (73.90)
Age	31-40	1 (4.40)
	41-50	2 (8.70)
	51-60	6 (26.10)
	61-70	9 (39.10)
	71-80	5 (21.70)
Education	Illiterate	3 (13.10)
	Below diploma	13 (56.50)
	Diploma	5 (21.70)
	Bachelor’s degree	2 (8.70)
Marital status	Single	0 (0)
	Married	20 (87)
	Widowed	3 (13)
Occupation	Unemployed	7 (30.40)
	Clerk	0 (0)
	Self-employed	3 (13.10)
	Retired	11 (47.80)
	Disabled	2 (8.70)
Living condition	Alone	0 (0)
	With spouse	5 (21.70)
	With Family	18 (78.30)
Ejection Fraction	10%	8 (34.80)
	15%	3 (13)
	20%	7 (30.40)
	25%	3 (13)
	30%	2 (8.80)

In the fourth stage of this cycle, the results of quantitative evaluation showed that the number of hospitalizations and length of hospital stay, 30 days after the implementation of the program, was significantly reduced compared to 30 days before the implementation of the program (P<0.001). The results are presented in Table 4.

**Table 4:** Comparison of the number of hospitalizations and length of hospital stay of heart failure patients 30 days before and 30 days after implementation of the discharge plan

Variable	Measuring status	Median	IQR^a^	Z Test (n=23)	P value*
Number of hospitalizations	Pre-test	1	(1-2)	-4.12	<0.001
	Post-test	0	(0-1)		
Length of hospital stay (days)	Pre-test	8	(6-13)	-4.06	<0.001
Post-test	0	(0-3)			

* Wilcoxon test;

a Interquartile Range

Results of qualitative evaluation showed the satisfaction of patients and health care providers with the way the program was implemented. Participants mentioned that it could significantly help the patients follow the orders more carefully and, therefore, avoid re-hospitalization. Also, discussions highlighted some shortcomings in the program, and the predominant themes were: a) lack of a uniform booklet for patients to record symptoms and medications, b) lack of coordinating visits based on the patients’ addresses and getting stuck in traffic, c) long wait in emergency department for critically ill patients who have referred to the hospital based on the treating physicians’ order, and d) lack of free phone line in the office for telephone follow-ups. 

The findings of the fifth stage showed that the participants believed although the discharge plan was running well and some results showed that the number of hospitalizations and length of hospital stay had significantly decreased, the results of qualitative evaluation showed that there were problems that could be solved by planning. 

In the sixth stage, based on the discussion and exchange of views, new decisions and solutions were adopted to solve the problems raised in the fifth stage. New decisions were preparing a booklet for registering the symptoms and the drugs used, coordinating the visit of patients who are on the same route in one day, designing special cards for patients who refer to hospital emergency department upon the advice of the nurse and the treating physician and are in the red zone of heart failure, and requesting a free phone line. 

Findings of the second cycle: In the first stage of this cycle, the participants proposed that in order to improve the discharge plan, collaboration among participants is necessary to make changes to facilitate the implementation of the program, and the participants’ responsibilities for change were identified.

And in the second stage, re-planning was done to reform and complete the program, and parts of the discharge plan were rewritten. Participants coordinated new changes as follow:

A booklet for registering the symptoms and the drugs used was prepared. This way it would be easier for the research team to monitor the patient’s condition.

A list of patients’ names and addresses was prepared by collaboration of the researcher, nurses and the hospital’s transport manager. Then, it was decided that from now on, the visit of patients who are on the same route would be coordinated in one day.

Special cards were designed by the care team for patients who refer to hospital emergency department upon the advice of the nurse and the treating physician and are in the red zone of heart failure, to be assigned immediately with no waste of time. In this regard, coordination was made among the participants and staff of emergency department.

After negotiations with the center’s research assistant, a free telephone line was provided to the office room.

In the third stage, 71 home visits and 285 telephone follow-ups were performed. In the fourth stage of the second cycle, the final evaluation of the discharge plan showed that the number of hospitalizations and length of hospital stay significantly decreased 3 months after implementation of the discharge plan (P<0.001). Also, the self-care behavior of the patients, 3 months before and after the implementation of discharge plan, was significantly different (P<0.001) and self-care was improved. The results are presented in Table 5.

**Table 5:** Comparison of the number of hospitalizations, length of hospital stay, and self-care behavior of heart failure patients 3 months before and 3 months after implementation of the discharge plan

Variable	Measuring Status	Median	IQR^a^	Z Test (n=23)	P value*
Number of hospitalizations	Pre-test	2	(1-3)	-4.30	<0.001
Post-test	0	(0-1)		
Length of hospital stay (days)	Pre-test	16	(9-19)	-4.11	<0.001
Post-test	0	(0-5)			
Self-care behavior	Pre-test	38	(35-43)	-4.17	<0.001
Post-test	18	(17-22)

* Wilcoxon test;

a Interquartile Range

In the fifth stage of the second cycle, predominant themes of the reflection in the group discussion session were as follows: Participants involved in the study believed that the reduction in hospital admissions was significant, most patients were in a safe area of heart failure after implementation of the discharge plan and treatment adherence was improved with the given training. 

Finally, in the sixth stage of the second cycle, suggestions were made to upgrade the program. These suggestions include the following: 

Allocation of fixed personnel in the implementation of patient discharge plan, possibility of prescribing injectable drugs (requiring monitoring at home), promotion of job motivation in service providers in this service department, development of guidelines for home nursing services for patients with heart failure to facilitate the clinical judgment of nurses, employment of nursing staff in this service department, and establishment of a home care unit affiliated with the hospital.

## DISCUSSION

The aim of this study was to improve heart failure patients’ discharge plan through action research. In this study, we reported the development and implementation of a discharge plan for heart failure patients which reduced hospitalization and was effective in improving the patients’ self-care by improving collaboration among healthcare professionals.

This project was a learning process through which stakeholders were empowered. Not only patients’ outcomes improved, but also healthcare professionals were empowered in different aspects of heart failure management. Collaboration among stakeholders facilitated the change in the context. In this regard, another study indicated that to improve the patient experience and the quality of discharge communications, strong health team communication and integrated processes are required, which leads to improved post-discharge outcomes. ^23^

In the present study, the discharge plan was designed and implemented in three areas of patient empowerment, telephone follow-up, and home visit after discharge. The educational needs of patients were identified, and then appropriate educational content was prepared. All heart failure readmission reduction programs include patient education as a key component. ^24^ All steps of the study were implemented with collaboration of the care team and hospital managers. Researchers stated that empowerment in health was a process with the interaction of the individual and the environment, by which the individual’s control over decision-making and performance affected the health more. ^25^ Nurses are an important part of the heart failure health care system, and they play a vital role in improving the patients’ health literacy and empowerment. ^26^

Implementation of a telephonic transitional care intervention within 48-72 hours of hospital discharge can reduce 30-day hospital readmission of heart failure patients. ^27^ The findings of a systematic review study in patients with heart failure and chronic obstructive pulmonary disease showed that early follow-up of patients reduced hospitalization 30 days after discharge. ^28^ In the present study, we considered early follow-up too, which began 24 hours after their discharge. Another study showed that discharge guidance with telephone follow-up resulted in better therapeutic adherence and decrease in re-hospitalization and death rates in heart failure patients. ^29^

Participants in the present study believed that following patients after discharge by home visit could increase the success rate of the discharge plan and reduce re-hospitalization. In this regard, results of another study showed that home visits reduced the re-admission of patients with complex chronic disease within 30 days after discharge. ^30^ It is necessary to make changes in Iran’s health care programs and pay attention to patient follow-up and home visits after discharge. ^31^

Having a structured and systematic discharge system is necessary to facilitate the discharge process and improve the patients’ clinical and social health results. Effective discharge plan reduces re-hospitalization and increases the quality of care. ^32^ Results of a study also pointed out the need for a complete discharge plan and stated that for better results and condition, a comprehensive and complete discharge readiness for heart failure patients and their families was essential. ^33^ The present study showed that the implementation of the designed discharge plan improved the self-care behavior of the patients. The patient was trained from the time of hospitalization during several training sessions, and it continued in all stages of home visits and telephone follow-ups. Improving self-care can be attributed to long-term and frequent training. In this regard, another study also concluded that a proper discharge plan, including educating patients with schizophrenia and their families and following up on discharge, led to improved patient self-care. ^34^ Furthermore, the results of a study showed that the implementation of the discharge plan for patients undergoing coronary artery bypass grafting increased the patients’ satisfaction with nursing care and improved their self-care ability. ^35^ Heart failure patients with effective self-care experience fewer deaths and hospitalizations. Self-care is the process of maintaining health by promoting health-promoting behaviors, monitoring, and managing the disease symptoms. Self-care is very important in the management of heart failure patients. ^36^ By reviewing the patient’s readiness for discharge and implementing the standard discharge plan, re-hospitalization can be prevented and giving more information to the patient can increase the quality of the discharge process. ^37^

The present study was a major attempt to improve discharge plan and patient outcomes. The important point of the present study’s discharge plan was the two-way communication between the patient and the care team, which enabled follow up continuously and for a long time. Collaboration among relevant stakeholders in all the stages of the two cycles of this action research facilitated change in the discharge plan.

This study was an innovation in the clinical context, but it should be noted that action research requires much time and energy due to its participatory nature. Coordinating group meetings was sometimes difficult because the participants had different roles and responsibilities at the center, and it was difficult to hold meetings at a time when everyone could attend.

One of the limitations of our study was the time limit for implementing more than two cycles of the research and the lack of financial and human resources in the implementation of the program.

## CONCLUSION

In this study, high readmission rate of the heart failure patients was a problem, and the solution was found in improving heart failure patients’ discharge plan through action research, which resulted in reduced re-hospitalization and improved self-care behavior. It can be concluded that collaboration of stakeholders in designing and implementing healthcare programs can facilitate change and improve patient outcomes.
